# Telerehabilitation Policy Report: Interprofessional Policy Principles and Priorities

**DOI:** 10.5195/ijt.2024.6687

**Published:** 2025-01-15

**Authors:** Evelyn Abrahante Terrell, Andy Bopp, Josh Krantz, Kim Karr, Steve Kline, Kristen Neville, Tammy Richmond, Kyle Zebley

**Affiliations:** 1 Nicklaus Children's Health System, Miami, Florida, USA; 2 American Occupational Therapy Association, Bethesda, Maryland, USA; 3 American Speech-Language-Hearing Association, Rockville, Maryland, USA; 4 American Physical Therapy Association, Alexandria, Virginia, USA; 5 Go 2 Care, Los Angeles, California, USA; 6 American Telemedicine Association, Arlington, Virginia, USA

**Keywords:** Advocacy, Audiology, Public Health Emergency, Occupational therapy, Physical therapy, Policy, Telehealth, Telepractice, Speech therapy

## Abstract

While the public health emergency is over, telehealth and telepractice will continue to play a pivotal role in supporting health equity for diverse and vulnerable individuals in underserved communities. The American Occupational Therapy Association, the American Physical Therapy Association, the American Speech-Language-Hearing Association, and the American Telemedicine Association are professional associations representing the interests of more than 564,000 rehabilitation services professionals in the United States. These organizations have shared priorities including legislative, regulatory, and state-based advocacy efforts. Continued advocacy and promotion by professional organizations, providers and patients alike is necessary to ensure that all rehabilitation providers are included in any telehealth lists of federally and state approved providers. In addition, key health care industry stakeholders want telehealth to be a permanent option for care delivery and ensure that telehealth will remain widely available to support health equity and access to critical rehabilitation services for all.

During the COVID-19 pandemic, telehealth demonstrated its clear value in ensuring access to safe, affordable, and quality healthcare delivered in an efficient manner. The global pandemic left an enormous impact on rehabilitation service delivery and how health care organizations, providers, professional associations, and their members have implemented omnichannel care models that include in-person and virtual care. While the public health emergency (PHE) is over, telehealth and telepractice will continue to play a pivotal role in supporting health equity for diverse and vulnerable individuals in underserved communities.

Rehabilitation service professionals include occupational therapists (OTs), occupational therapy assistants (OTAs), physical therapists (PTs), PT assistants (PTAs), speech-language pathologists (SLPs), audiologists, and other members of the care team who serve individuals across the lifespan in various health care settings, facilities, and specialty areas. Rehabilitation services delivered via telehealth reduce geographic and access barriers and address a critical workforce shortage of professionals. Virtual care has been shown to improve patient satisfaction and experience and can help to address health equity challenges; these are paramount to ensuring optimal outcomes and access to quality services for all people. (Bican et al., 2021; Krasovsky et al., 2021; Little et al., 2021).

Science-driven, evidence-based telehealth interventions by rehabilitation service professionals have enabled people of all ages to develop, regain, and build functional communication, mobility, and independence in everyday activities, enabling them to live life to its fullest. Telehealth has improved access to timely therapy services and has been a lifeline for individuals with chronic conditions and those residing in rural or underserved areas, lacking transportation, or facing health care disparities or other socioeconomic barriers. In use cases, some benefits of therapy services provided via telehealth cannot be replicated in a facility or office setting. For example, occupational therapy practitioners report that home safety issues can be identified and addressed when a patient or caregiver provides visual access to the patient's home through telehealth technologies. This is critical for fall prevention, evaluating potential for decreasing activities of daily living performance function, engaging in patient education and avoiding emergency room visits and hospital admissions, which all have the potential of reducing the cost of care.

The American Occupational Therapy Association (AOTA), the American Physical Therapy Association (APTA), the American Speech-Language-Hearing Association (ASHA) and the American Telemedicine Association (ATA) are professional associations representing the interests of more than 564,000 rehabilitation services professionals in the US. AOTA, APTA, ASHA, and the ATA have shared priorities including legislative, regulatory, and state-based advocacy efforts, and the organizations collaborate with policymakers to support legislation that will promote access to care and ensure sustainability of telehealth services.

As a result of the collaborative advocacy of the AOTA, APTA, ASHA, the ATA and others, key telehealth policies were passed or expanded by the federal government in 2023 and 2024:

- Therapy practitioners have continued access to remote therapeutic monitoring (RTM) codes which were added in 2022 to the Medicare Part B Physician Fee Schedule.
- The Centers for Medicare & Medicaid Services (CMS) confirmed continuation of coverage for telehealth outpatient therapy services delivered by OT, PT, SLP and other professionals who remain on the list of distant site practitioners to serve Medicare beneficiaries in the home setting via telehealth or telecommunications technologies. Practitioners may continue to bill for telehealth services under the Medicare Physician Fee Schedule when furnished remotely in the same way as during the PHE.
- Additional services were added to the Medicare telehealth services list, increasing caregiver access to skill assessment and training via telehealth.

The American Relief Act, 2025 (H.R. 10545), signed into law on December 21, 2024, extends key telehealth flexibilities through March 31, 2025, including waiving geographic and originating site restrictions (such as the patient's home) and allowing therapists to deliver telehealth services across care settings. These provisions build on the Telehealth Beyond COVID-19 Act (H.R. 4040), enacted in late 2022, which provided a two-year extension of Medicare telehealth flexibilities through the end of 2024, ensuring continuity of access after the Public Health Emergency ended on May 11, 2023.

AOTA, APTA, ASHA, and the ATA have collaborated to develop, support, or endorse legislation to assist members in sustaining telehealth omnichannel care models that include in-person and virtual care, with focus on specific priorities including:

- Federal and state level advocacy to advance permanent telehealth reform.
- Removal of restrictions on provider type, supporting enhanced provider autonomy, and ensuring access to non-physician providers.
- Promote lifting of service restrictions in federal law such as limiting specific technologies, modality of care types, and support audio-only services.
- Permanently removing restrictions on where the patient must be located to receive health care access, and support provisions that would enable the patient's home to serve as the originating site for telehealth.
- Support the bipartisan Expanded Telehealth Access Act of 2023 (H.R. 3875/S.2880 in the 118^th^ Congress) which would permanently expand the scope of practitioners eligible to provide telehealth services. This legislation would add audiologists, OTs, PTs, and SLPs as permanent authorized providers of telehealth under the Medicare program.
- Endorse the Telehealth Modernization Act (H.R.7623/S.3967 in the 118^th^ Congress) which is a comprehensive telehealth bill that includes a provision to establish OTs, PTs, SLPs, and audiologists as permanent Medicare telehealth providers by removing the waiver sunset in statute.
- Support continued reimbursement of telehealth services delivered by facility-based providers under Medicare Part B—including hospital outpatient departments, skilled nursing facilities, and rehabilitation agencies—consistent with congressional intent under the Consolidated Appropriations Act of 2023 (ASHA, 2023).
- Support licensure compacts and portability to enable practice across state borders as well as advocating in support of expanding telehealth authorization by state licensing boards.
- Support coverage by Medicaid and commercial insurers of therapy and audiology services provided via telehealth.
- Advocate to relax requirements related to remote monitoring including both remote physiologic monitoring and remote therapeutic monitoring in the CY2024 Physician Fee Schedule Proposed Rule, specifically related to the 16-day monitoring requirement over a 30-day period that may impact access to services.
- Supporting fair payment for telehealth services (AOTA, 2023a, 2023b; APTA, 2023; ASHA, 2023; ATA, 2022a, 2022b).

Congressional action is critical to avoid a “Telehealth Cliff” for audiology and physical, occupational and speech language therapy services. If Congress does not permanently expand the scope of practitioners eligible to provide telehealth services or extend the current authority that enables rehabilitation services professionals to furnish services in this manner beyond March 31, 2025, when current waivers enacted by Congress will expire, access to care would be abruptly cut-off. Medicare beneficiaries would lose access to critical virtual care options that have become a lifeline to many, especially for underserved and rural populations (ATA, 2023a).

Congressional action is also imperative to facilitate the continued advancement of telehealth innovation by rehabilitation services professionals to optimize patient engagement and outcomes. Continued advocacy and promotion by professional organizations, providers and patients alike is necessary to ensure that all rehabilitation providers are included in any telehealth lists of federally and state approved providers. In addition, key health care industry stakeholders want to make telehealth a permanent option for care delivery and ensure that telehealth will remain widely available to support health equity and access to critical rehabilitation services for all.
